# Racial and Ethnic Disparities in Cesarean Delivery and Indications Among Nulliparous, Term, Singleton, Vertex Women

**DOI:** 10.1007/s40615-021-01057-w

**Published:** 2021-07-12

**Authors:** Ijeoma C. Okwandu, Meredith Anderson, Debbie Postlethwaite, Aida Shirazi, Sandra Torrente

**Affiliations:** 1 Department of Obstetrics & Gynecology, Kaiser Permanente San Francisco, San Francisco, CA USA; 2 Division of Research, Kaiser Permanente Northern California, Oakland, CA USA; 3 Department of Graduate Medical Education, Kaiser San Francisco, San Francisco, CA USA

**Keywords:** Cesarean delivery, Obstetric outcomes, Disparities

## Abstract

**Objective:**

To compare cesarean delivery rates and indications by race/ethnicity among nulliparous women with term, singleton, vertex presentation deliveries.

**Methods:**

This is a retrospective cohort study of nulliparous women delivering term, singleton, vertex neonates at Kaiser Permanente Northern California from 1/1/2016 to 6/30/2017. Women with cesarean for elective, malpresentation, or previa were excluded. Multivariable logistic regression models adjusting for maternal, neonatal, and facility factors were used to assess the likelihood of cesarean by race/ethnicity. Further modeling was performed to examine odds of cesarean for the indications of failure to progress and fetal intolerance by race/ethnicity.

**Results:**

The cohort of 16,587 racially/ethnically diverse women meeting inclusion and exclusion criteria consisted of 41.62% White, 27.73% Asian, 22.11% Hispanic, 5.32% Black, and 3.21% multiple race/other women. In adjusted logistic regression models, all race and ethnic categories had higher odds of cesarean deliveries in comparison to White women. Black women had the highest odds of cesarean delivery (adjusted OR [aOR] = 1.73, 95% CI: 1.45–2.06), followed by Asian (aOR = 1.59, 95% CI: 1.45–2.06), multiple race/other (aOR = 1.45, 95% CI: 1.17–1.80), and Hispanic (aOR = 1.43, 95% CI: 1.28–1.59) women. Compared with White women, Asian (aOR = 1.46, 95% CI: 1.22–1.74) and Hispanic (aOR = 1.25, 95% CI: 1.03–1.52) women had higher odds of failure to progress as the indication. Among women with failure to progress, Black (aOR = 0.50, 95% CI: 0.30–0.81), Hispanic (aOR = 0.68, 95% CI: 0.53–0.87), and Asian (aOR = 0.77, 95% CI: 0.61–0.96) women were less likely than White women to reach 10 cm dilation. Compared with White women, Black women were more likely to have cesarean delivery for fetal intolerance (aOR = 1.51, 95% CI: 1.10–2.07). Among women with fetal intolerance of labor, there were no significant differences by race/ethnicity for Apgar score or neonatal intensive care unit admission.

**Conclusions:**

Race/ethnicity was significantly associated with the odds of cesarean and indication. All other race/ethnicity groups had higher odds of cesarean compared with White women. Compared with White women, Black women had greater odds of fetal intolerance as an indication, while Hispanic and Asian women had greater odds of failure to progress. Maternal, neonate, and facility factors for cesarean delivery did not explain the observed disparities in cesarean delivery rates.

## Introduction

Cesarean delivery is the most common surgical procedure for women in the United States (U.S.) [1]. Of four million U.S. deliveries per year, one in three is delivered by cesarean [1]. Women undergoing cesarean delivery are exposed to the inherent risks of surgery, maternal morbidity and mortality, and adverse neonatal outcomes [2]. In the United States, nulliparous, term, singleton, vertex (NTSV) cesarean delivery has increased from 18.4% in 1997 to 26.9% in 2013, with rates 5% greater in non-Hispanic Black women and 1% greater in Hispanic women compared with non-Hispanic White women [3]. The racial/ethnic differences in cesarean delivery rates have persisted over time, even for women considered at low risk for cesarean delivery [3].

In 2015, Black women in California had the highest NTSV cesarean delivery rate (29.5%), compared to White (24.6%), Hispanic/Latina (25.1%), and Asian (25.6%) women [4]. Disparate NTSV cesarean delivery rates are a public health concern which raises the question of potential overuse. Cesarean deliveries are associated with risk of surgical complications, increased obstetrical related costs, greater likelihood of delivery by cesarean in subsequent births, and risk abnormal placentation and hysterectomy [2]. Potential reasons for the observed disparity include differences in maternal characteristics such as age, diabetes and hypertension, obesity, and need for induction of labor [5, 6]. However, it is also essential to recognize the potential influence of race on health outcomes. Race itself is not biological but rather a social construct with profound socioeconomic and health consequences [7, 8].

The current body of research confirms that racial disparities in NTSV cesarean delivery exist. However, prior studies are limited by including patients with variable insurance and the inability to adjust for access to care during pregnancy. The existing literature has no consensus to explain the observed racial and ethnic disparities in cesarean delivery. A California-based population study of women’s childbirth experiences points to the potential role of maternity care practices in cesarean delivery rates (e.g., labor induction, the timing of admission during labor, and use of epidural) [9]. Other studies hypothesize differences may be attributed to socioeconomic status, preference, poor health access, and low participation in obstetric care [10–12].

Prior studies have not explicitly evaluated potential race/ethnic differences in labor patterns. The purpose of this study is to assess the extent to which maternal race and ethnicity are associated with the rate of cesarean delivery, indication for cesarean delivery, and labor patterns among NTSV women within an integrated health care system, Kaiser Permanente Northern California (KNPC), where insurance and access factors should be equitable.

## Methods

### Study Design

**Fig. 1:**
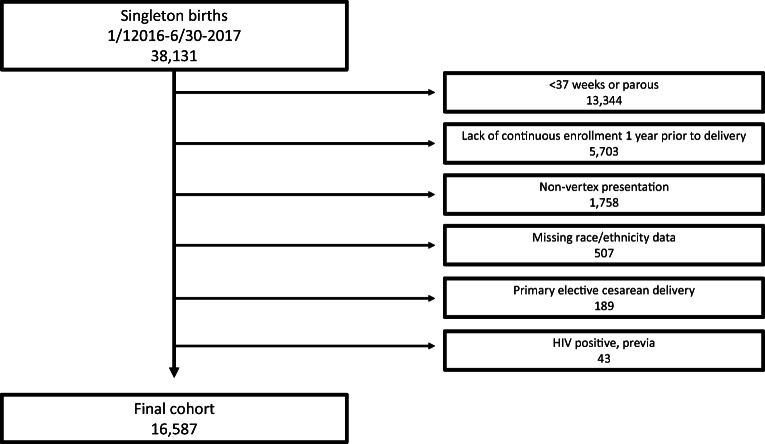
Cohort study flow diagram showing patient selection and exclusion criteria and the number of patients included in the study analysis

A retrospective cohort study of all NTSV deliveries at KPNC hospitals between January 1, 2016, and June 30, 2017, was conducted. A total of 16,824 NTSV deliveries were identified in the study period, 16,587 (98.59%) of which met inclusion criteria. The 237 women excluded consisted of 189 women with a primary elective cesarean, 38 women with a cesarean indication of placenta previa, and ten women with a cesarean for breech presentation (Fig. 1).

To reduce the confounding effect of unequal access to preconception and prenatal care, only women who were KPNC members for at least 1 year before delivery were included. As part of the health system’s standard of care, all obstetric patients had a standardized prenatal intake recorded in the electronic medical record (EMR). Women were excluded if they delivered before 37 weeks gestation, delivered a non-viable fetus, had a previous birth, had a multiple gestation pregnancy, had a scheduled elective cesarean, or had a medical condition that may have precluded vaginal delivery (e.g., non-vertex presentation, placenta, or vasa previa human immunodeficiency virus [HIV] positive status).

The primary outcomes were (1) mode of delivery (cesarean delivery) and (2) indication for cesarean delivery. The primary predictor was maternal race/ethnicity obtained from the EMR. Race/ethnicity data is strictly self-reported and not clinician reported. The data is gathered from questionnaires administered to patients during clinic visits and hospitalizations. Race/ethnicity data is grouped into Asian, Black, Hawaiian, multiracial, American Indian/Alaska Native, Pacific Islander, unknown/other, and White categories. Hispanic ethnicity is categorized as Hispanic Yes/No. For this study, race/ethnicity was further collapsed into the following five categories: Hispanic, non-Hispanic White, Black, Asian (includes Hawaiian and Pacific Islander), and multiple/other races (includes American Indian and Alaska Native). All women who identified as Hispanic were categorized as Hispanic, regardless of race. Women with missing race data but with Hispanic ethnicity were classified as Hispanic. Because race/ethnicity is a significant focus of this study, women with missing race and missing ethnicity were excluded. Non-Hispanic White, Black, Asian, and multiple race categories will be referred to as White, Black, Asian, and multiple races for the remainder of the manuscript. The rates of NTSV cesarean delivery and indications are reported by race/ethnic group.

Secondary outcomes were assessed by performing sub-analyses on cesarean delivery indications: failure to progress or fetal intolerance of labor. For the purposes of this study, we were interested in only the primary indication for cesarean delivery; thus, for cases in which there were multiple indications or the indication for cesarean delivery was unclear, a chart review was performed to determine the primary clinical indication. Among women with failure to progress, the odds of achieving complete dilation by race/ethnicity were assessed. Among women with fetal intolerance of labor as an indication, we compared mean Apgar score and odds of NICU admission by race/ethnicity. Other demographic and clinical characteristics such as maternal age at delivery, body mass index (BMI), self-reported maternal education level and household income, marital status, presence of midwifery service during labor and delivery, maternal medical history, obstetric history and procedures, gestational age at birth, and birth weight were electronically extracted from the EMR. Data on cesarean delivery indications and time spent in labor were electronically extracted from KPNC labor and delivery records.

### Study Setting

Kaiser Permanente is one of the nation’s largest fully integrated health systems consisting of a nonprofit insurance plan, hospital and clinics, and a physician group. In 2016, KPNC facilities included 18 medical centers and nearly 250 medical offices; the 4.5 million membership is demographically representative of the Northern California population. The health care system focuses on preventative medicine facilitated by a fully integrated electronic medical record, regional standardization, and guideline-based care. In obstetrics specifically, standardization of care is reflected in the prenatal checklist implemented throughout all facilities in KPNC. The prenatal checklist specifies recommendations for frequency of visits, timing of tests and screening, and home monitoring for patients with co-morbidities such as hypertension and diabetes. Fifteen of the twenty-one hospitals offer labor and delivery services, and KPNC operates three obstetrics and gynecology residency programs. All sites have readily available in-house obstetricians and anesthesiologists who work in a shift model, and in all but two hospitals, the care team also includes certified midwives.

### Hypothesis

Based on 2015 California birth data, the most significant difference in NTSV cesarean delivery rate was between White and Black women, 24.6% and 29.5%, respectively [13]. We expected that our health system would have similar rates as the general population of California. Thus, we hypothesized that 25% of NTSV White and 30% or more of Black women in the KPNC insured population would have cesarean deliveries. A two-group chi-square test with a 0.05 two-sided significance level and 80% power to detect the difference between the two groups required 3835 White and 733 Black women (total sample size of 4568).

### Statistical Methods

Univariate statistics were used to describe the demographic and clinical maternal and neonatal characteristics of the cohort. Bivariate analyses were conducted to compare these characteristics by race/ethnicity and identify possible predictors for cesarean delivery. To assess the extent to which type of indication for cesarean delivery impacted neonatal outcomes, mean neonatal Apgar scores of women who underwent cesarean delivery for indication of fetal intolerance of labor were compared with those who had a cesarean delivery for another indication. To accomplish this, ANOVA and Tukey’s test were employed. Comparisons involving categorical variables were performed using chi-square or Fisher’s exact tests. Normally distributed continuous variables were compared using Student’s t-tests or analysis of variance (ANOVA), and comparisons of non-normally distributed continuous variables employed the Wilcoxon rank-sum or Kruskal-Wallis test. The study period’s cesarean rate was calculated for the cohort by race/ethnicity, and rates are reported as percentages. Frequencies and proportions of each cesarean indication by racial/ethnic group were calculated. The amount of time in labor before cesarean delivery was compared by racial/ethnic group.

The association of the primary dichotomous outcome of cesarean delivery with maternal race/ethnicity was examined in unadjusted logistic regression models with White women as the reference group. Multivariable models were adjusted for risk factors that were clinically relevant or found to be significantly associated with cesarean delivery in bivariate analysis. Final models for the three outcomes, cesarean delivery, as well as fetal intolerance of labor and failure to progress among women who had a cesarean delivery, were adjusted for maternal age at delivery, income, education, marital status, induction of labor, availability of midwifery care at birth hospital, gestational age, and neonate birth weight. Maternal medical conditions, obesity, gestational diabetes, gestational hypertension, chronic hypertension, and pre-eclampsia, were also adjusted for. Pre-eclampsia was included in the multivariate model as it independently is not an indication for cesarean delivery. Associations of fetal intolerance to labor and failure to progress as dichotomous outcome variables in unadjusted and adjusted logistic regression models by race/ethnicity were examined. In the adjusted logistic regression models, operative vaginal delivery was combined with vaginal delivery into one category.

Using multivariable linear regression, the differences in mean length of the second stage (hours) before cesarean delivery were compared by race/ethnicity with White women as the reference group. All univariate, bivariate, and multivariable analyses were performed using Statistical Analysis Systems (SAS) 9.3; p-values of <0.05 were considered significant. This data-only study was approved with a waiver of consent by the Kaiser Foundation Research Institute’s Institutional Research Board.

## Results

Demographic and socioeconomic characteristics of the women by race/ethnicity are reported in Table 1. The racial/ethnic composition of the cohort was as follows: 41.62% White, 27.73% Asian, 22.11% Hispanic, 5.33% Black, and 3.21% multiple races. Overall, 68.31% of the cohort delivered vaginally, 9.18% had an operative vaginal delivery, and 22.47% delivered by cesarean (data not known). Maternal age, education, marital status, and income were significantly associated with race/ethnicity.

**Table 1:** Maternal demographic and socioeconomic factors by race/ethnicity

Characteristic	Race/ethnicity					P-value^*^
	Asian/Hawaiian/Pacific Islander n = 4599 (27.73%)	Black n = 883 (5.32%)	Hispanic n = 3668 (22.11%)	Multiple Races/American Indian/Alaskan Native n = 533 (3.21%)	White n = 6904 (41.62%)	
Age (years), Mean (SD)^*^	31.23 (4.35)	26.61 (6.11)	27.06 (5.96)	29.01 (5.82)	30.40 (5.11)	<0.0001^**†**^
Age (years)						
Less than 20	34 (0.74)	94 (10.65)	371 (10.11)	32 (6.00)	160 (2.32)	<0.0001
20–24	234 (5.09)	304 (34.43)	1044 (28.46)	98 (18.39)	788 (11.41)	
25–29	1238 (26.92)	201 (22.76)	942 (25.68)	142 (26.64)	1786 (25.87)	
30–34	2154 (46.84)	177 (20.05)	869 (23.69)	168 (31.52)	2792 (40.44)	
35–39	799 (17.37)	81 (9.17)	363 (9.90)	74 (13.88)	1156 (16.74)	
≥40	140 (3.04)	26 (2.94)	79 (2.15)	19 (3.56)	222 (3.22)	
Marital status						
Married/partnered	3634 (79.02)	263 (29.78)	1856 (50.60)	328 (61.54)	5052 (73.17)	<0.0001
Single/never married	795 (17.29)	587 (66.48)	1734 (47.27)	194 (36.40)	1665 (24.12)	
Divorced/separated/widowed	20 (0.43)	13 (1.47)	27 (0.74)	3 (0.56)	46 (0.67)	
Other/unknown	150 (3.26)	20 (2.27)	51 (1.39)	8 (1.50)	141 (2.04)	
Income (annual)						
<$40,000	494 (10.74)	308 (34.88)	1092 (29.77)	89 (16.70)	794 (11.50)	<0.0001
$40,000–$79,999	724 (15.74)	157 (17.78)	728 (19.85)	105 (19.70)	1146 (16.60)	
$80,000 and above	2366 (51.45)	134 (15.18)	688 (18.76)	184 (34.52)	3259 (47.20)	
Declined or missing	1015 (22.07)	284 (32.16)	1160 (31.62)	155 (29.08)	1705 (24.70)	
Education						
Some high school or below	37 (0.80)	37 (4.19)	171 (4.66)	16 (3.00)	59 (0.85)	<0.0001
High school graduate or GED	173 (3.76)	133 (15.06)	542 (14.78)	46 (8.63)	382 (5.53)	
Some college or technical school	551 (11.98)	263 (29.78)	1032 (28.14)	109 (20.45)	1332 (19.29)	
Completed college or graduate school	2983 (64.86)	207 (23.44)	984 (26.83)	224 (42.03)	3584 (51.91)	
Unmarked/missing	855 (18.59)	243 (27.52)	939 (25.60)	138 (25.89)	1547 (22.41)	

^*^P-value for the Chi-squared Test of Association unless otherwise noted

^**†**^F-test for equality of group means

The cesarean delivery rate for all NTSV women in the cohort was 22.47% (3727/16,587). Race/ethnicity was significantly associated with delivery (p < 0.0001) (Table 1). White women comprised 41.62% of the cohort but accounted for 37.51% of cesarean deliveries performed. On the other hand, Asian and Black women comprised lower proportions of the cohort (27.73% and 5.33%, respectively) than their total cesarean deliveries (29.54% and 6.20%, respectively). Table 2 shows maternal, perinatal, and labor and system-level factors by race/ethnicity. As seen, all factors investigated were significantly associated with race/ethnicity.

**Table 2:** Maternal, perinatal, and labor and system level factors by race/ethnicity

Characteristic	Race/ethnicity					P-value^*^
	Asian/Hawaiian/Pacific n = 4599 (27.73%)	Black n = 883 (5.32%)	Hispanic n = 3668 (22.11%)	Multiple Races/American Indian/Alaskan Native n = 533 (3.21%)	White n = 6904 (41.62%)	
Maternal factors						
Obesity at prenatal intake (BMI ≥ 30)						
Yes	473 (10.28)	309 (34.99)	1146 (31.24)	136 (25.52)	1394 (20.19)	<0.0001
No	4126 (89.72)	574 (65.01)	2522 (68.76)	397 (74.48)	5510 (79.81)	
Gestational diabetes						
Yes	519 (11.29)	39 (4.42)	245 (6.68)	35 (6.57)	295 (4.27)	<0.0001
No	4080 (88.71)	844 (95.58)	3423 (93.32)	498 (93.43)	6609 (95.73)	
Chronic or gestational hypertension or pre-eclampsia						
Yes	367 (7.98)	127 (14.38)	350 (9.54)	58 (10.88)	781 (11.31)	<0.0001
No	4232 (92.02)	756 (85.62)	3318 (90.46)	475 (89.12)	6123 (88.69)	
Chorioamnionitis						
Yes	62 (1.35)	4 (0.45)	40 (1.09)	5 (0.94)	42 (0.61)	0.0005
No	4537 (98.65)	879 (99.55)	3628 (98.91)	528 (99.06)	6862 (99.39)	
Perinatal factors						
Gestational age (days), Mean (SD)	277.09 (7.68)	277.56 (8.00)	277.89 (7.81)	277.79 (7.76)	279.47 (8.02)	<0.0001^**†**^
Neonate birth weight (grams), Mean (SD)	3246 (437)	3303 (463)	3388 (450)	3403 (468)	3457 (448)	<0.0001^**†**^
Intrauterine growth restriction						
Yes	68 (1.48)	17 (1.93)	28 (0.76)	4 (0.75)	76 (1.10)	0.006
No	4531 (98.52)	866 (98.07)	3640 (99.24)	529 (99.25)	6828 (98.90)	
Fetal macrosomia						
Yes	61 (1.33)	17 (1.93)	86 (2.34)	16 (3.00)	169 (2.45)	0.0004
No	4538 (98.67)	866 (98.07)	3582 (97.66)	517 (97.00)	6735 (97.55)	
Labor and system level factors						
Induction of labor						
Yes	996 (21.66)	221 (25.03)	859 (23.42)	107 (20.08)	1701 (24.64)	0.001
No	3603 (78.34)	662 (74.97)	2809 (76.58)	426 (79.92)	5203 (75.36)	
Availability of midwifery service (during labor and delivery)						
Yes	4025 (87.52)	829 (93.88)	3230 (88.06)	489 (91.74)	6105 (88.43)	<0.0001
No	574 (12.48)	54 (6.12)	438 (11.94)	44 (8.26)	799 (11.57)	
Delivery type						
Spontaneous vaginal	2841 (61.77)	607 (68.74)	2586 (70.50)	356 (66.79)	4947 (71.65)	<0.0001
Assisted vaginal	657 (14.29)	45 (5.10)	215 (5.86)	47 (8.82)	559 (8.10)	
Cesarean	1101 (23.94)	231 (26.16)	867 (23.64)	130 (24.39)	1398 (20.25)	

^*^P-value for the Chi-squared Test of Association unless otherwise noted

^**†**^F-test for equality of group means

**Fig. 2:**
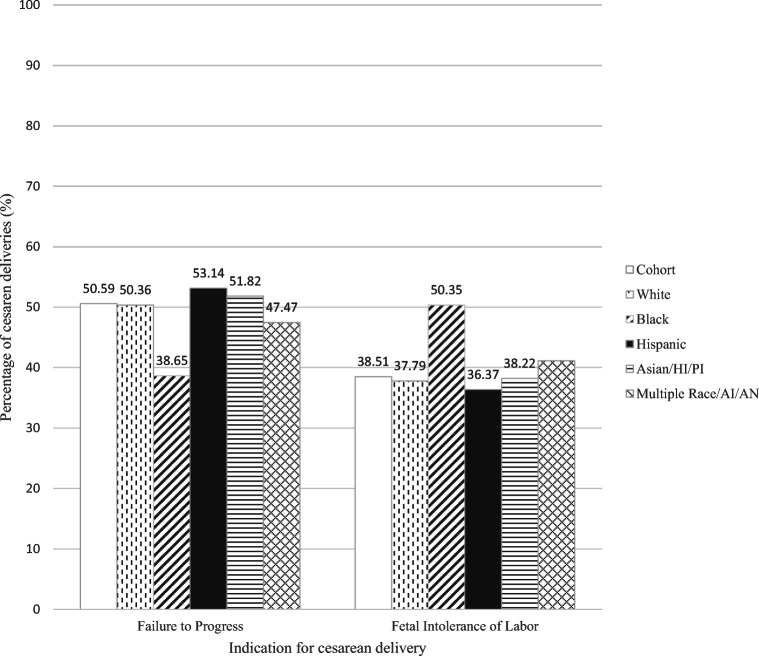
Proportions of cesarean deliveries with indication of failure to progress or intolerance of labor, by race/ethnicity. Shown are the two most common indications for cesarean delivery by race/ethnicity for the study population (n = 16,587). Hispanic group showed greatest percentage for failure to progress but lowest percentage for fetal intolerance of labor. The Black population showed the reverse: greatest percentage for fetal intolerance of labor and lowest percentage for failure to progress as indication

Figure 2 shows the two most common indications for cesarean delivery by race/ethnicity. For all race/ethnicity groups combined, the most common cesarean delivery indications were failure to progress (50.59%) and fetal intolerance of labor (38.51%). Of the 3727 women who had CS in the cohort, 486 (13.02%) had both failure to progress and fetal intolerance of labor. Of these women, 475 had either failure to progress or fetal intolerance of labor as the primary indication; 11 had failure to progress and fetal intolerance of labor, but neither listed as the primary indication (in these cases, chart review as performed to determine the primary indication). Some indications were very rare (e.g., active HSV, failed instrumentation, malpresentation, and cord prolapse); thus, only the most common indications were the focus of analysis. Indication type was significantly associated with race/ethnicity. Of women who had cesarean deliveries, a higher percentage of Black women had a cesarean for fetal intolerance of labor (50.35%) compared to White (37.79%) and Asian (38.22%), Hispanic (36.37%), or multiple race women (41.14%) (Fig. 2).

In the adjusted logistic regression model, all race and ethnic categories had higher odds of cesarean deliveries compared with White women. Black women had the highest odds of cesarean delivery (adjusted OR [aOR] = 1.73, 95% CI: 1.45–2.06), followed by Asian (aOR = 1.59, 95% CI: 1.45–2.06), multiple race/other (aOR = 1.45, 95% CI: 1.17–1.80), and Hispanic (aOR = 1.43, 95% CI: 1.28–1.59) women (Table 3).

**Table 3:** Adjusted odds ratios of cesarean delivery, fetal intolerance of labor, and failure to progress by race/ethnicity controlling for demographic and clinical characteristics

	Cesarean Delivery (n = 16,587)	Fetal intolerance of labor^*^ (n = 3727)	Failure to Progress^*^ (n = 3727)
Race/ethnicity (ref: White)	aOR^†^ (95% CI^‡^)	aOR (95% CI)	aOR (95% CI)
Asian/Hawaiian/Pacific Islander	1.59 (1.44–1.76)	0.90 (0.76–1.07)	1.46 (1.22–1.74)
Black	1.73 (1.45–2.06)	1.51 (1.10–2.07)	0.77 (0.56–1.04)
Hispanic	1.43 (1.28–1.59)	0.87 (0.72–1.05)	1.25 (1.03–1.52)
Multiple races/American Indian/Alaskan Native	1.45 (1.17–1.80)	1.27 (0.86–1.86)	1.03 (0.70–1.51)

^*^Among women who had cesarean deliveries, 486 (13.02%) of the women who had cesarean deliveries had both fetal intolerance of labor and failure to progress as indications

^†^Adjusted odds ratio from logistic regression models adjusted for maternal age at delivery, income, education, marital status, obesity, gestational diabetes, gestational or chronic hypertension or preeclampsia, induction of labor, availability of midwifery services, gestational age, and neonate birth weight

^‡^Confidence interval

Adjusted logistic regression models showed that the odds of cesarean delivery for fetal intolerance of labor for Black women were approximately 50% higher than for White women (aOR 1.51, 95% CI: 1.10–2.07) (Table 3). Asian (aOR = 1.46, 95% CI: 1.22–1.74) and Hispanic (aOR = 1.25, 95% CI: 1.03–1.52) women had higher odds of cesarean delivery for the indication failure to progress compared with White women (Table 3). Among women who underwent cesarean delivery for the indication failure to progress, Black, Hispanic, and Asian women were less likely than White women to reach 10 cm dilation (aOR = 0.50, 95% CI 0.30–0.81; aOR = 0.68, 95% CI 0.53–0.87; aOR = 0.77, 95% CI 0.61–0.96, respectively) (data not shown).

Among women delivered by cesarean and reached the second stage of labor, adjusted multivariable linear regression results showed that Asian women had significantly longer mean in the second stage. In contrast, Black women had significantly shorter mean in the second stage than White women (Table 4).

**Table 4:** Adjusted^*^ linear regression parameter estimates for the length of the second stage of labor in hours prior to cesarean delivery by race/ethnicity controlling for demographic and clinical characteristics

	Length of the second stage of labor β	95% CI^†^	P-value^‡^
Race/Ethnicity (ref: White)^§^			
Asian/Hawaiian/Pacific Islander	0.45	(0.36 to 0.53)	<0.0001
Black	−0.27	(−0.43 to −0.10)	0.002
Hispanic	0.03	(−0.06 to 0.12)	0.52
Multiple races/American Indian/Alaskan Native	0.19	(0.00 to 0.38)	0.06
Intercept	−3.49	(−4.74 to −2.25)	<0.0001

^*^Adjusted mean differences from linear regression models adjusted for maternal age at delivery (ref: 25–29 years), income (ref: ≥$80,000), education (ref: Completed college or graduate school), marital status (ref: Single/Never Married), obesity (ref: No), gestational diabetes (ref: No), gestational or chronic hypertension or preeclampsia (ref: No), induction of labor (ref: No), availability of midwifery services (ref: No), gestational age, and neonate birth weight

^†^Confidence interval

^‡^P-value for the β coefficient from linear regression

^§^Ns for race/ethnicity categories in this analysis: Asian = 2705, Black = 480, Hispanic = 2231, Multiple races/AI/AN = 323, White = 4570

Finally, despite the greater likelihood of cesarean delivery for fetal intolerance of labor among Black women, pairwise comparisons of Black women to each of the other race/ethnicity groups showed no significant differences in mean Apgar scores at 1 or 5 min (data not shown). Neonatal intensive care unit admissions were not significantly associated with race/ethnicity among women with cesarean delivery for fetal intolerance of labor (data not shown).

## Discussion

### Principal Findings

In our examination of the association between maternal race/ethnicity association and route of delivery, data showed that women in all non-White race/ethnicity categories had higher odds of cesarean delivery compared with White women. Data also showed the indication for cesarean delivery was significantly associated with race/ethnicity. While Asian and Hispanic women had higher odds of cesarean delivery for the indication failure to progress than White women, Black women had higher odds of cesarean delivery for fetal intolerance of labor. Results also demonstrated that among women who underwent cesarean delivery for the indication of failure to progress, Black, Hispanic, and Asian women were less likely than White women to reach 10 cm dilation. Finally, analysis of the second stage of labor demonstrated Asian women had significantly longer mean in the second stage. In contrast, Black women had significantly shorter mean time in the second stage than White women.

In a racially and ethnically diverse cohort of 16,587, insured nulliparous women who delivered a live, vertex singleton infant within an integrated healthcare system, race/ethnicity was associated with the route of delivery. Like prior studies, we found that women of all race/ethnicity categories other than White had higher odds of cesarean deliveries compared to White women [14–19]. Despite adjustments for socioeconomic, medical, and obstetric risk factors in this insured population, the observed disparities persisted.

Similar to the findings of a study from the University of Massachusetts Memorial Medical Center (UMMMC) [15], we found that Asian and Black women had higher odds of cesarean delivery than White women. As we did, the authors of the UMMMC study concluded that racial and ethnic disparities persisted even among women considered to be at low risk for cesarean [15]. As well, we found that while Asian women on average had longer second stages of labor, they were more likely to undergo cesarean delivery for failure to progress.

Consistent with prior studies, our data showed that Black women are more likely to undergo cesarean delivery for fetal intolerance of labor. This indication has the potential for inter-clinician variability. In a retrospective cohort study, Stark et al. found that despite accounting for maternal, perinatal, and perinatal factors associated with cesarean delivery, Black women were more likely to undergo cesarean for non-reassuring fetal status [16]. Similarly, Bryant et al. found that Black women were 1.5 times more likely to undergo cesarean delivery than White women and were also more likely to undergo the procedure for the indication of non-reassuring fetal heart tracing [17], thus suggesting the possibility of other contributing factors in the process of shared decision making, such as the fears and influences that patients and their families may bring to the decision for cesarean delivery [20, 21]. Data from prior work demonstrate that the possibility a woman’s race/ethnicity may subconsciously affect the delivering provider’s clinical decision when the mode of delivery is not completely clear [22]. Conversely, a large multi-center observational study showed that Black, Asian, and Hispanic women have higher rates of cesarean delivery than White women; however, the difference in odds of cesarean could not be traced to the disparate application of guidelines for labor management [23].

Prior studies have attributed differences in cesarean delivery rates and their indications to variation in health insurance or maternal comorbidities [24, 25]. However, a study conducted at Kaiser Permanente Southern California found that compared with White women, the primary cesarean rate was 25% higher for Black women, 19% higher for Asian/Pacific Islander women, but 14% lower for Hispanic women, after adjusting for confounding factors [14]. The finding of lower cesarean deliveries in Hispanic women was not mirrored in our study; instead, our data showed Hispanic women had higher odds of cesarean delivery compared with White women (aOR = 1.42, 95% CI: 1.28–1.58). While the study mentioned above population was within a similar integrative health system, its cohort was inherently different from our study population, as it included preterm deliveries. It is also possible that the two Latinx populations’ countries of origin differed (e.g., Mexican and Puerto Rican).

Our study reduced the potential confounding effect of disparate access to preconception and prenatal care by including only women who were KPNC members for at least 1 year before delivery. Prior studies did not consider this key component in their analyses. Also, our study specifically evaluated potential race/ethnicity differences in labor patterns.

### Clinical and Research Implications

The KPNC healthcare system utilizes a prenatal checklist that provides clinical guidance for the frequency of prenatal appointments, indicated testing, interventions, and counseling for uncomplicated obstetric patients. This model provides structure and facilitates standardization of care; however, clinicians have the flexibility to alter the frequency of visits based on their assessment of need and risks. Because the hospital, insurance, and physicians are an inter-related system, physicians are not financially incentivized to perform cesarean deliveries. Given the broad access and standardization of care and removal of monetary incentives, the observed racial and ethnic disparities were surprising. Thus, our findings suggest the influence of unmeasured factors that may contribute to the observed racial/ethnic differences. Possible factors may include provider bias, gaps in patient-provider communication, time at which delivery occurred (e.g., at change of shift), staff racial and ethnic background not reflecting that of the patient population, and variation interpretation of fetal heart monitoring [26–30].

The department of Maternal and Child health at KPNC uses the National Institutes of Health and Child Development (NICHD) categories 1, 2, and 3 to interpret fetal heart rate tracings. The NICHD categories were defined to reduce the subjectivity of assessing fetal heart tracings, and clinicians use this with the clinical scenario to evaluate fetal distress. Prior studies have noted the poor interobserver and intraobserver reliability of fetal heart monitoring, particularly in the evaluation of variability and decelerations—key components in the assessment of fetal distress or intolerance of labor [30–32]. Although fetal heart monitoring interpretation relies on objective measures, the existence of interobserver reliability highlights some component of subjectivity. As any management is dependent on a subjective interpretation, the variance in interpretation ultimately leads to differences in management. Johnson et al. investigated the ability of fetal monitoring to predict fetal outcomes. The study found that fetal monitoring had consistently higher sensitivity, but both lower specificity and negative predictive value for Black women compared to White women [33]. As lower specificity results in higher incidence of false-positive results, the authors contend the inaccuracy in the prediction of fetal distress suggests that Black women may undergo interventions such as cesarean delivery at a higher rate than their White counterparts but with less likelihood that the clinical outcome will be affected. To that end, Hefland et al. studied physician interpretation of electronic fetal monitoring and found that physicians are more likely to perform a cesarean section on patients the provider perceived as high risk even when presented with the same fetal tracing as patients considered low risk [32]. Thus, management decisions then reflect the comfort level of the decision maker rather than a clear algorithm.

In our study, neonatal intensive care unit admissions and Apgar scores did not significantly differ between race/ethnic groups. This finding may mean that some groups are offered cesarean delivery more often than indicated. Alternatively, one could argue that surgical intervention occurred at the appropriate time, thus averting more cases of neonatal depression that correspond to lower Apgar scores. Healthcare uses tools such as NICHD to decrease subjectivity; however, as previously discussed, the art and practice of medicine inherently have some aspect of subjectivity [30–33]. Another possible reason for the observed difference in cesarean rates between groups could be variation in the burden of underlying medical comorbidities [32]. However, comorbidities were controlled for in the adjusted logistic regression model. Still, the significant impact of race/ethnicity on the odds of cesarean delivery and indications for cesarean delivery persisted in our study. Additionally, there exists the possibility that some mothers and families may be more inclined to accept cesarean delivery because they perceive it as better or safer care [20].

Given the potential influence of unmeasured factors that may have contributed to the disparities observed in this study, future research should consider investigating factors not previously studied in-depth, such as cultural perceptions, implicit bias, and patient-provider communication. Investigation of these topics is a potential opportunity to identify social determinants of health that influence obstetric outcomes. A better understanding of these phenomena can guide future interventions to achieve racial and ethnic health equity.

### Strengths and Limitations

There are some limitations of this study to consider. First, while the patient population is racially/ethnically representative of the geographic area it serves, findings may not be generalizable to communities in different geographic locations and race/ethnic composition (particularly where the proportion of Hispanic/Latinx population differs), uninsured women, or women not cared for within an integrated healthcare system. Second, while the women in our study self-reported their race/ethnicity, some categories were combined for analytic purposes. A third limitation is that during the study period, the delivery summary in which physicians and nurses recorded cesarean indication did not include whether the delivery occurred during the first or second labor stage. Thus, the indication of failure to progress included cesarean deliveries performed for inability to dilate (<10 cm dilation) and second stage arrest (10 cm dilation/pushing phase). The electronic delivery summary also did not indicate during which stage of labor a cesarean delivery for fetal intolerance of labor occurred. Having this information would provide a better understanding of what stage of labor the cesarean delivery occurred and explain if concern for fetal well-being tended to occur in earlier or later stages of labor. Another limitation is the presumption only people who identified as women gave birth in this cohort. During the study period, the EMR did not include information about gender expression outside of woman/female. Finally, our study is limited in that it does not include a measure to reflect provider bias that could account for observed disparities. This is a critical factor that should be the focus of future research.

This study had several strengths: first, the large cohort, consisting of over 16,000 low-risk nulliparous women who planned to have a vaginal delivery. Second, it was based within KPNC, an integrated healthcare system with a diverse insured patient population that is demographically representative of Northern California [34]. Prior studies have suggested that disparities in cesarean rates may be rooted in social circumstances such as insurance and access to care. We excluded women who were not members of KPNC for 1 year before pregnancy and the entirety of their pregnancy, thus minimizing potential confounders inherent in access to care and differences in prenatal care. In regression models, we controlled for medical and socioeconomic factors known to impact health outcomes, such as maternal education, age, and income level. Finally, while this is a retrospective study, records demonstrating inconsistencies were validated by the investigators by manual chart review.

## Conclusions

Our study provides a significant contribution to the knowledge of cesarean rates and indications by race/ethnicity among NTSV insured women. This data demonstrates that racial and ethnic disparities exist in the mode of delivery and indication of cesarean delivery among women considered low risk. These observed differences do not appear to be related to common risk factors. The information obtained in this study may indicate the potential impact of racism and bias on health outcomes. The increasing rate of cesarean deliveries in the United States is a significant public health problem that disparately affects women of different races and ethnicities. An understanding and recognition of the existence of disparities are the first steps to addressing the pervasive inequities in maternal outcomes. The extent of existing literature is limited on how implicit bias may affect health care outcomes through direct and indirect pathways. Furthermore, studies evaluating the role of implicit bias of care providers, patient self-advocacy, and patient-provider communication on the delivery outcome are needed.

Our findings provide further support for the American College of Obstetricians and Gynecologists consensus statement encouraging healthcare providers and health systems to identify existing race/ethnic disparities in maternal outcomes and employ strategies and resources that can be used to address differences to achieve safe and equitable healthcare for all childbearing women [18]. Current quality guidelines for assessing the number of NTSV cesarean deliveries do not require stratification of race and ethnicity. This study underscores the phenomenon that while a health care system may meet overall NTSV quality measures, sub-group analysis may show otherwise. Hospitals and health care systems interested in addressing obstetric health inequities may consider such a study at a local level and implementing supplemental prenatal support and programs, specifically (i.e., doulas as patient advocates) for women of color.
